# Obstacles to prior art searching by the trilateral patent offices: empirical evidence from International Search Reports

**DOI:** 10.1007/s11192-016-1858-9

**Published:** 2016-04-07

**Authors:** Tetsuo Wada

**Affiliations:** Faculty of Economics, Gakushuin University, 1-5-1 Mejiro, Toshima-ku, Tokyo, 171-8588 Japan

**Keywords:** Examiner citations, International Search Report (ISR), International patent families, Patent Cooperation Treaty (PCT), Prior art search, Triadic patents

## Abstract

Despite many empirical studies having been carried out on examiner patent citations, few have scrutinized the obstacles to prior art searching when adding patent citations during patent prosecution at patent offices. This analysis takes advantage of the longitudinal gap between an International Search Report (ISR) as required by the Patent Cooperation Treaty (PCT) and subsequent national examination procedures. We investigate whether several kinds of distance actually affect the probability that prior art is detected at the time of an ISR; this occurs much earlier than in national phase examinations. Based on triadic PCT applications between 2002 and 2005 for the trilateral patent offices (the European Patent Office, the US Patent and Trademark Office, and the Japan Patent Office) and their family-level citations made by the trilateral offices, we find evidence that geographical distance negatively affects the probability of capture of prior patents in an ISR. In addition, the technological complexity of an application negatively affects the probability of capture, whereas the volume of forward citations of prior art affects it positively. These results demonstrate the presence of obstacles to searching at patent offices, and suggest ways to design work sharing by patent offices, such that the duplication of search costs arises only when patent office search horizons overlap.

## Introduction

Patent citations have been widely utilized as empirical tools for studies of patent systems, particularly in relation to economic value and knowledge flow (Trajtenberg 1990; Jaffe et al. 1993; Hall et al. 2005). Although earlier studies did not distinguish between examiner and applicant citations, subsequent studies have examined whether they differ. For example, a study by Alcacer and Gittelman (2006) demonstrated the similarity between examiner and inventor citations with respect to geographical distance. While follow-up work has compared examiner and applicant citations with respect to other dimensions of patent systems, including their relationship with renewal rates (Hegde and Sampat 2009) and the probability of use for rejections (Cotropia et al. 2013), few have analyzed how patent offices are influenced by obstacles to prior art searching. Given that examiners (and searchers working for patent offices) can never be faultless in conducting prior art searches, the types and extent of any obstacles should form part of the policy design parameters.

For example, the Patent Prosecution Highway (PPH) programs allow a patent office to utilize previous search and examination work from an earlier prosecution process at a participating patent office, provided that patent applications are made in the two countries with the same priority date, and with a corresponding (i.e., substantially the same) set of claims. The premise of this program is to expedite prosecution at the later-acting office by way of utilizing information gathered in the earlier examination. Theoretically, a patent office examiner is supposed to search for relevant prior art worldwide to confirm novelty.^1^ If (in an ideal case) the outcome of a search reliably covers all relevant prior art, subsequent patent prosecution processes in different countries can simply utilize the search outcome without a duplicate search. In other words, a single office can act as a search agent for all other offices when there is no impediment to searching.

However, if patent offices have very different local advantages in technological knowledge, then the searches are not duplicates, such that searches at later-acting offices need to be conducted on top of any search outcomes supplied by the other offices. Accordingly, in order to design an international work-sharing plan between offices, the types and extent of any obstacles to prior art searching should be scrutinized to reduce duplicate search costs (or obtain a more complete search through overlapping searches) through collaboration. Unfortunately, we know little about how such obstacles stand in the way of searchers and examiners.^2^ It would then be useful to first define and test several possible searching “distances” for use at patent offices when searching for prior art.

One reason why there has been no large-scale study to date on the obstacles to prior art searching by patent offices is a lack of measurement. To address this, we employ International Search Reports (ISRs) to measure the search difficulties of the trilateral patent offices, and test how “distance” binds officials, including both geographical distance along with similar kinds of obstacles to prior art searching, without relying on comparison with applicant citations. In conducting the analysis, we also consider applicant self-selection, given applicants from both the US and Japan can choose the European Patent Office (EPO) as their search agency, where the EPO has a reputation for quality prior art searching (such that applicants seeking a more stringent search may select the EPO ex ante).

## Background and prior literature

Following pioneering work in measuring the effects of knowledge spillover through patenting data (Jaffe 1986) and the value of patents through patent citations (Trajtenberg 1990), Jaffe et al. (1993) and Jaffe and Trajtenberg (1999) considered the measurement of knowledge diffusion by patent citation. They found that knowledge diffusion is geographically localized, assuming that patent citations show traces of knowledge transmission. However, while survey results confirm that patent citations indicate knowledge flow with considerable noise (Jaffe et al. 2000; Duguet and MacGarvie 2005), there is criticism of the method in that patent citations are often unrelated to knowledge transfer between inventors. This is partly because patent citations include examiner citations, and because even attorneys acting on behalf of inventors in preparation for patent prosecution sometimes add applicant citations.

Given that examiners are not inventing and because the perceptions of inventors regarding prior knowledge have been a central concern for innovation research, one area of recent research is how “noisy” examiner citations are in relation to applicant citations. For example, Alcacer and Gittelman (2006) has found that examiner and applicant citations have similar distributions in terms of the geographical distance between citing and cited patents. Their results, as based on US patent data, partly contradict those from EPO data, although geographical distance also binds examiner citations by the EPO (Criscuolo and Verspagen 2008). There are other advantages of examiner citations for economic research, including in terms of better measuring the value of patents than by applicant/inventor citations (Hegde and Sampat 2009). Other detailed comparisons between applicant citations and examination citations have revealed that examiners do not rely on applicant-submitted information on prior art (Cotropia et al. 2013). However, these studies did not consider examiner citations independently from applicant/inventor citations.

In their search for prior art, examiners are professionals, but they are not perfect. Recent micro-level studies on examiner experience level and granting behavior (Lemley and Sampat 2012; Frakes and Wasserman 2014) as well as others on examiner citations (Cotropia et al. 2013) acknowledge the limitations of examiners. However, apart from a few studies, the economics literature has not considered the extent to which obstacles to searching bind examiners. A related series of research in Melbourne (Jensen et al. 2005; Webster et al. 2007; Palangkaraya et al. 2011; Webster et al. 2014) compared the results of patent grants from the trilateral offices and concluded that patent offices are biased toward local applicants (and against foreign applicants) in terms of patent grants. While differential grant rates against foreign applicants can be caused by “prejudiced” examinations in each office, examiner bias (i.e., local advantages in technological knowledge) may also contribute to the seemingly differential rates of patent grants. A remaining question is how we can measure examiner bias as caused by obstacles to searching.

Most of these existing studies use patent citation data from either a single country or two regions at most. Each data set of examiner citations in a country show only the results of a single patent office. However, if we combine multiregional citation data and consolidate citations pairs through international patent families, we should obtain in principle a way to measure the difference between regions with respect to the same criterion of family-to-family citation. That is, patents in an international family cite patents in another international patent family. Put differently, given examiner citations across different regions show traces of the examination outcomes in each region, we can track back and compare how examiners behave when citing the same prior art. As explained in the following section, we assume that every examiner citation in the national phase could have been added in its earlier ISR phase if there are no obstacles to searching by examiners, or searchers for an International Search Authority (ISA). Drawing on this assumption and the concept of family-to-family citation, we can statistically evaluate the obstacles to searching.

## The methodology: PCT and ISR as the basis for empirical measurement

The measurement of examiner search obstacles is itself an impediment to research on examiners and searchers at patent offices. We propose a method of measuring search obstacles of the trilateral patent offices by focusing on ISRs issued by different ISAs, specifically the patent offices in Europe, the US, and Japan, according to the Patent Cooperation Treaty (PCT).

**Fig. 1:**
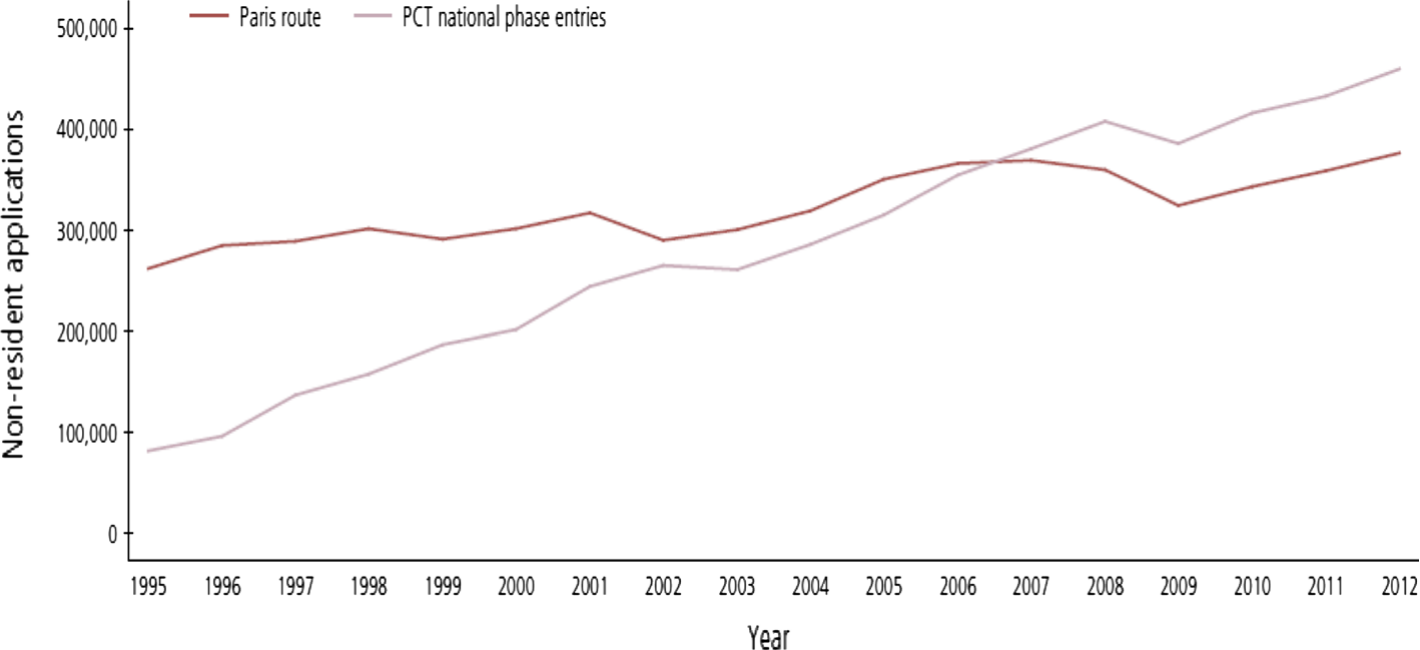
Nonresident PCT and Paris Convention route entries (WIPO 2011, p. 48)

Before explaining the details of citation-level methodology, we note that PCT applications are increasingly important for applicants seeking patent protections internationally, and that PCT applications should receive more attention from the field of scientometrics. The number of PCT national phase entries from abroad has already surpassed the number of nonresident applications via the Paris Convention route worldwide (Fig. 1). While the PCT is now the *main route* for international applications, there have been few empirical studies of the PCT system. Given that the trilateral offices—the EPO, the US Patent and Trademark Office (USPTO), and the Japan Patent Office (JPO)—received most PCT applications before the mid-2000s, it is reasonable to limit our sample to those PCT applications made to and examined by all of the three offices, at least up to 2005.^3^

An ISA gives a PCT application received at a patent office an ISR at the time of international publication of the application. Under the PCT, “…an applicant must file an application with a Receiving Office (RO) and choose an international searching authority to provide an International Search Report and a written opinion on the potential patentability of the invention” (WIPO 2011). An ISR contains a list of prior arts, and the set of prior arts becomes part of the citations. ISRs are issued under a common search criterion established by the World Intellectual Property Organization (WIPO) under the PCT system. “The applicant generally has at least 30 months from the filing (priority) date to decide whether to enter the national phase in the countries or regions in which protection is sought” (WIPO 2011). The WIPO guidelines apply to every ISA when issuing an ISR, whereas some countries permit applicants to choose between ISAs. The same criteria for a prior art search apply for different patent offices, while national phase examinations do not have such standardized rules. We can then distinguish between cited patents added in the national phase by designated offices (or *DO*-*citedpatents*) and those cited patents caught earlier during the ISR (or *ISR*-*cited patents*).

**Fig. 2:**
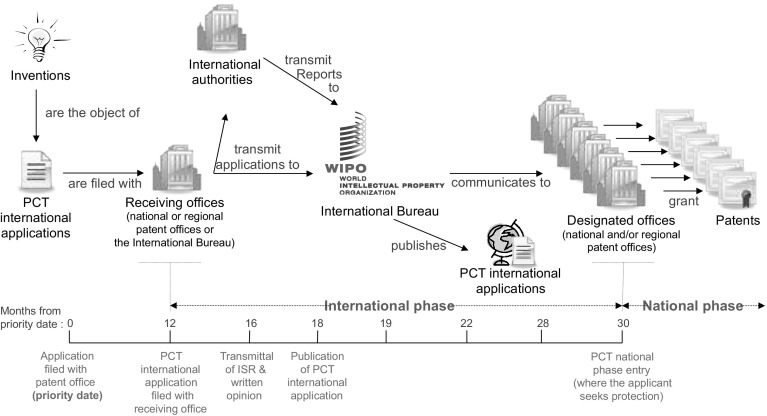
PCT procedure (WIPO 2011, p. 13)

As shown in Fig. 2, there are time differences between ISRs and national phase examinations, implying the existence of a lag between ISR-citations and DO-citations on average in the national phase. While ISRs are produced at an early stage, more searches occur later in national offices. Given that knowledge is geographically localized (Jaffe et al. 1993; Jaffe and Trajtenberg 1999), and knowledge diffusion takes time, the additional time between the ISR and the national phase search facilitates a more complete search in the later stage. We limit our sample to PCT applications examined in all three of the trilateral offices, meaning that any localized knowledge captured in any of these areas at the time of the ISR can be caught by the offices in the national phase in a less localized way.

**Fig. 3:**
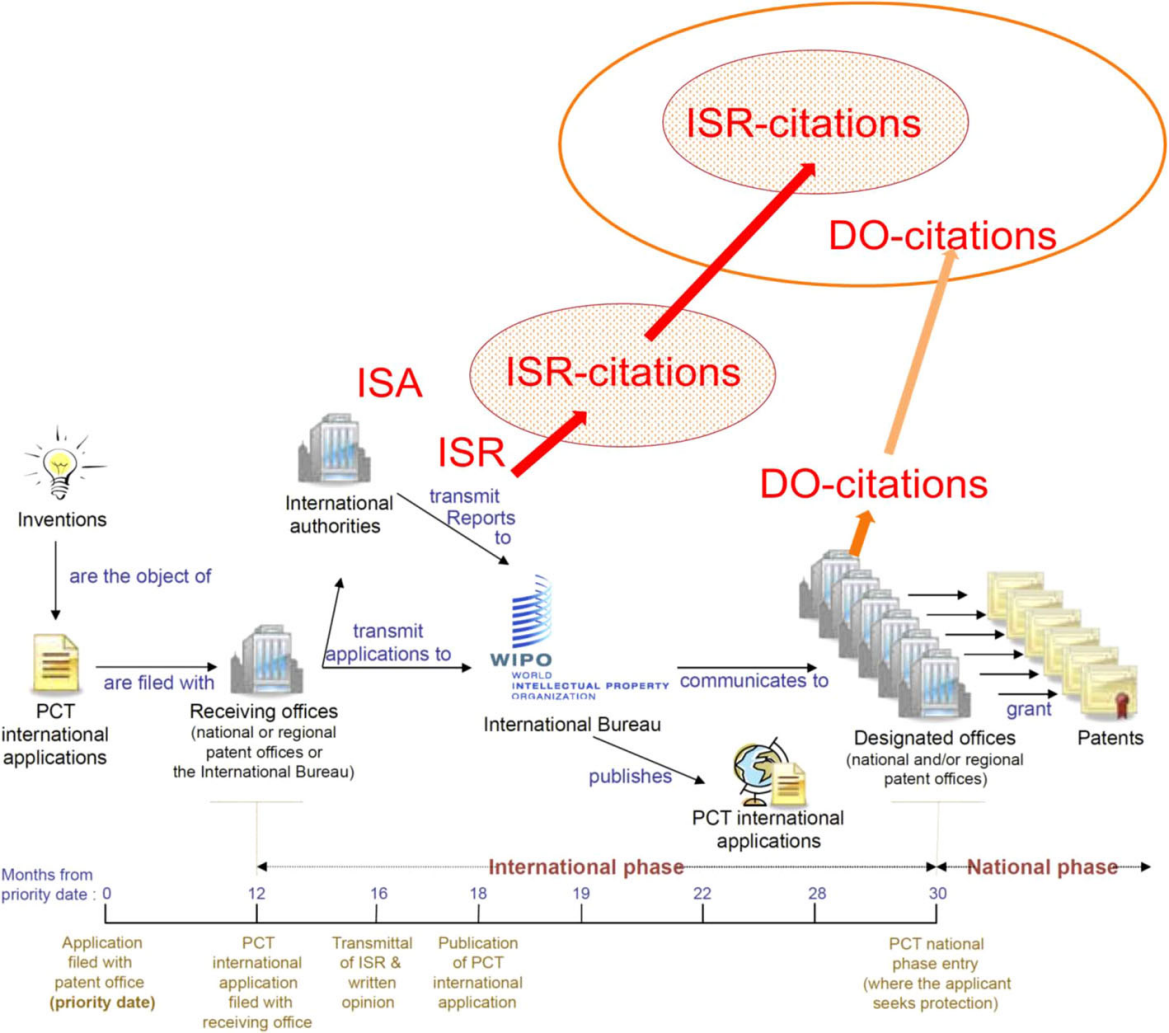
Dependent variable *found_in_ISR:* a binary variable representing the probability of a DO-citation or ISR-citation already included in the set of ISR-citations (modification to Fig. 2)

Following the logic above, we retrospectively define the probability of every cited patent for a PCT application (the union set of ISR-cited and DO-cited patents), consolidated and identified at the INPADOC family^4^ level, as already caught in the ISR of the originating PCT application (whether or not included in the ISR-cited patents) (Fig. 3). Taking this probability (*found_in_ISR*) as the dependent variable, we implement PROBIT analyses at the INPADOC family level with explanatory variables representing the various “distances” between citing and cited patents, including the technological complexity of the originating applications and other related indicators. Simply put, we assume that every DO-cited patent for a PCT application has been cited in its ISR if every citer and cited pair is consolidated at the INPADOC family level, and if examiners (or searchers for an ISA) are unbounded in their searching capability.

We should mention several caveats concerning the methodology. First, we exclude applicant (inventor) citations from the analysis because our primary objective is to evaluate the determinants of search completeness by the ISAs. However, when an applicant is relatively capable in searching for prior art, ex ante disclosure might affect the quality of a search by the patent office. To address this, we conduct additional analysis to consider the self-selection to the EPO of US and Japanese applicants. This is because the EPO has a reputation for a higher examination standard and therefore higher capability applicants from the US and Japan may choose the EPO as their ISA.^5^

Second, if a relevant prior art was missed at the time of an ISR, we assume that one of the designated offices (DOs) will cite it. In reality, DO-citations vary according to the different standards in different regions. Given that the US patent system does not provide citation category (such as “X” and “Y”) information, we have been unable to apply the same standard of rejection for a cited piece of prior art. In addition, DOs can never be perfect in a prior art search. Citations made by post-grant oppositions are included, but citations by post-grant litigations are not. Thus, the union set of DO-citations is only an approximation of the quasi-complete search made possible ex post. Conversely, DOs may cite prior art in response to an applicant action such as an amendment of claim, divisional application, or continuation. Although ISAs are supposed to cite prior art reasonably expected to be relevant in subsequent changes of claims, we cannot ex ante search all prior arts triggered by the ex post amendment of claims. Then, we may violate our basic assumption that “every DO-cited patent for a PCT application should have been cited in its ISR if ISAs are perfect in their search capabilities” if the amendment of claims is too drastic.

Third, sometimes outsourced to non-PTO agencies, we consider ISRs as the basis of evaluating PTOs because issuance is under the name of the patent office, not any private search agencies. We also consider only those citations made by the trilateral offices, such that search completeness made possible by nontrilateral offices is not considered.

Finally, given that PATSAT, our primary data source, records nonpatent literature in a nonstandardized format, we could not consolidate it across different records. For this reason, we only employ patent citations. Further, US citations are not as complete in PATSTAT. In particular, there is no record of citations for rejected applications in PATSTAT.^6^ Although it is usually possible to retrieve citations data from the Public PAIR database for rejected applications filed after 2001, we have been unable to combine the data from the two sources.

## Hypotheses

Given that ISR searchers (including examiners and searchers working for patent offices) are affected by obstacles to searching because of various “distances,” we hypothesize that a prior patent (found in the ISR or national phase) is more likely to be included in the ISR when distances are less problematic, i.e., if:

### **H1**

A relevant prior patent is geographically closer (shorter geographical distance).

### **H2**

A relevant prior patent is older (more knowledge diffusion time).

### **H3**

A relevant prior patent is from the same applicant (less organizational distance).

### **H4**

A relevant prior patent has a greater number of forward citations (more knowledge diffusion beforehand).

### **H5**

An application for which an ISR is issued has less scope, a lower number of claims, a fewer number of inventors, and a smaller size of international family (less complexity relative to search).

**Fig. 4:**
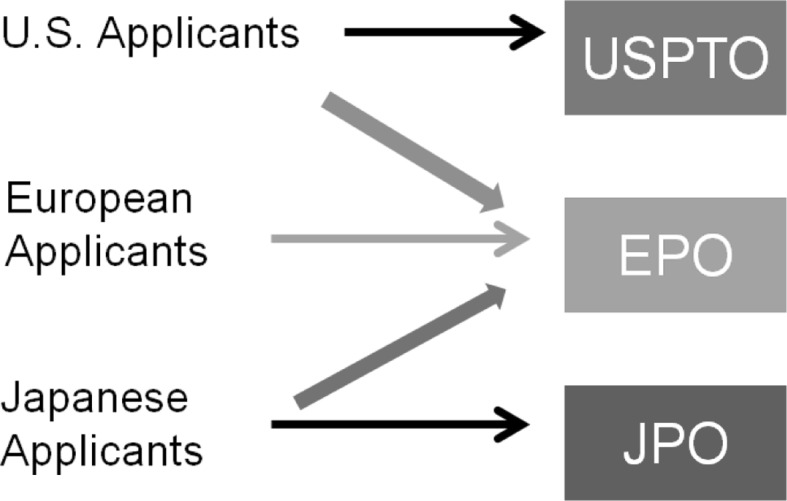
Selection of ISA from the RO

In addition, we consider the possibility that an applicant’s self-selection of an ISA affects the outcome variable. As shown in Fig. 4, PCT applicants from the US and Japan are permitted to select the EPO as their ISA, unlike applicants from European Patent Convention (EPC) contracting states who are not permitted to select the USPTO or the JPO as their ISA.^7^ Given the reputation of the EPO for high-quality prior art searches, applicants from the US and Japan may self-select if they seek a more stringent search at the EPO. We therefore include switching behavior on PCT applications for ISRs as one of the factors for ISR completeness, and use instrument variables for ISA-switch (a binary variable *ISA_changed*).

## The data source

The empirical domain of analysis is triadic patent applications through PCT with an earliest priority date within its international family between 2002 and 2005. Triadic PCT patent applications are defined here as INPADOC families that contain all EPO, USPTO, and JPO applications recorded on EPO’s PATSTAT database, with only one “WO” (PCT) application in a family. This means that a single PCT application initiates the international phase for all applications in a family. There are 97,828 international families used in the analysis. Although international applications to and from China and Korea have increased dramatically in the last 10 years, the trilateral patent offices of the EPO, the USPTO, and the JPO represent the vast majority of applications before 2005, which is our observation period.

We use EPO PATSTAT (2013 OCT version), and INPADOC family is the unit of analysis. Therefore, the accuracy of international families depends entirely on the INPADOC family table on PATSTAT. The citation data are also from PATSTAT (2013 OCT), and the JPO citation data is augmented using the Seiri–Hyojunka data (the standardized patent prosecution data of the JPO). We consolidate applicant identifiers using the EEE-PPAT database developed by ECOOM (Du Plessis et al. 2009; Magerman et al. 2009; Peeters et al. 2009).

As discussed, US citation data are not complete in PATSTAT because it does not record citations for rejected applications. Even after the publication rule change in the US in 2001, a published application went unrecorded in PATSTAT if the application was abandoned (possibly due to rejection). The lack of US citations for rejected applications may affect the results of our analysis, but we have not yet verified this.

**Fig. 5:**
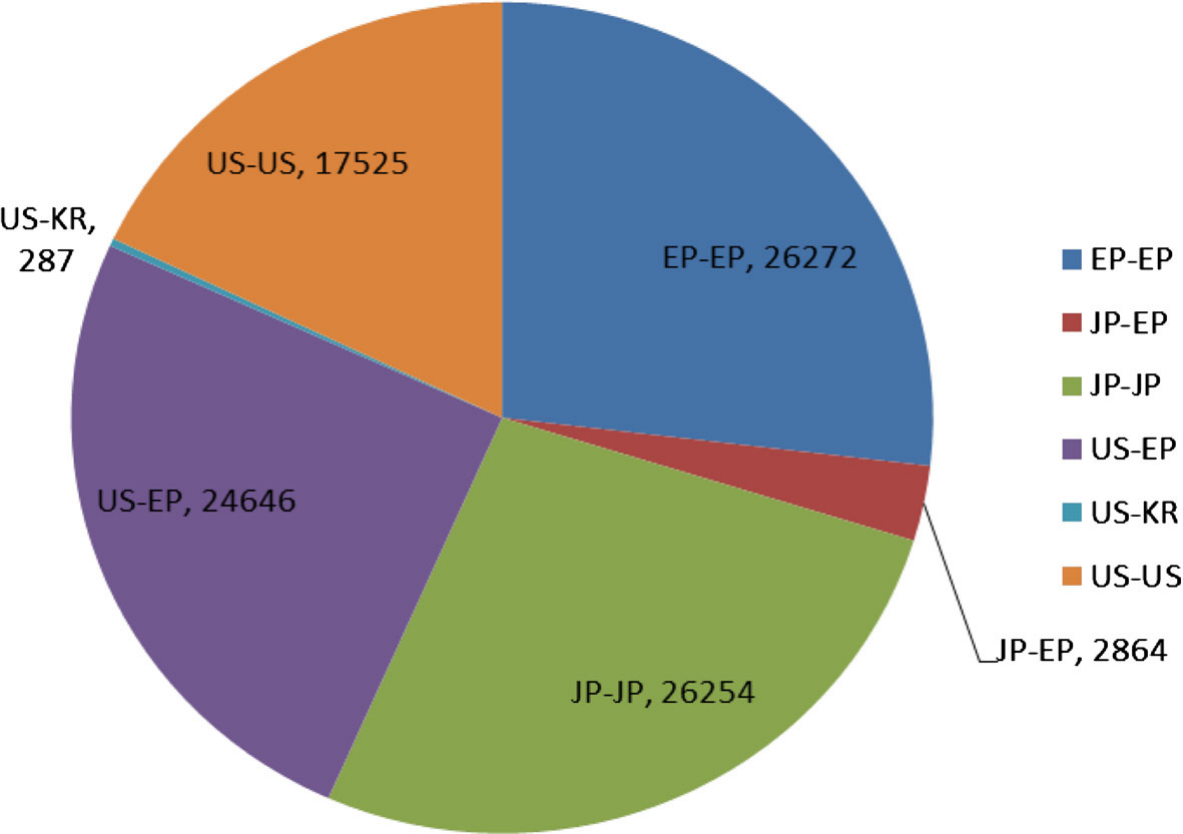
Composition of triadic PCT applications, priority years 2002–2005

Based on the data set described, applications from the EPO area represent more than a quarter of the entire sample, as shown in Fig. 5. In the figure, “JP-EP” denotes the JPO as the RO and the EPO as the ISA, and US-EP is the USPTO as the RO and the EPO as the ISA. Applicants from the EPO area are not permitted to choose ISAs; in contrast, US applicants are allowed to choose ISAs from the EPO, IP Australia, the Korean Intellectual Property Office (KIPO), the Rospatent (Russian Patent Office), etc. In fact, more than half of all PCT applications from the US choose the EPO as their ISA, while just 0.7 % select the KIPO.^8^ Applicants from Japan are allowed to choose either the JPO or the EPO as their ISA, but only about one-tenth of Japanese PCT applications have chosen the EPO as their ISA.

**Fig. 6:**
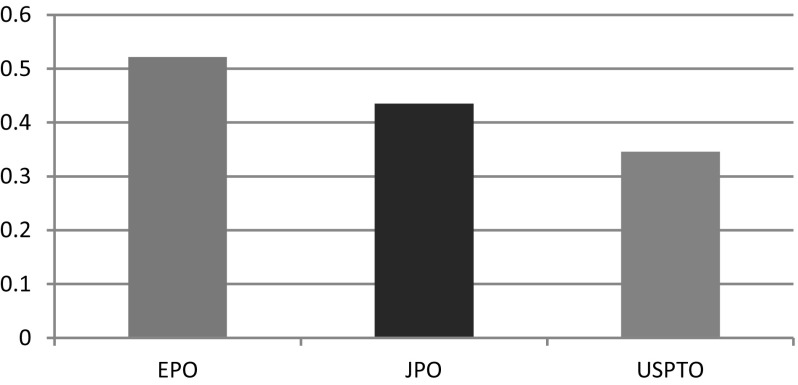
Simple average of the dependent variable *found_in_ISR* according to ISA

Underlying the selection of ISAs across the trilateral offices, there are differences in their reputations regarding completeness of search reports, i.e., the EPO has the best reputation. This is consistent with a simple comparison with the average of *found_in_ISR* for the three ISAs in Fig. 6. Given that the EPO has a good reputation, and given that applicants from the US or Japan can choose the ISA, we expect that self-selection by applicants influences the outcome variable, *found_in_ISR*. This is partly because applicants with inventions of higher economic value or with higher capability would spend more for a prior art search themselves, so that they would identify more prior art before submitting a formal application. Furthermore, highly capable applicants may desire a more stringent search in this early stage to avoid rejection in a later stage, i.e., the national phase. Indeed, there is evidence that applicants know that the EPO produces higher quality ISRs in general; the fees it charges are also relatively higher than those of the other offices.^9^ In order to account for self-selection, we hypothesize that the more experienced and capable an applicant in the US or Japan is in terms of technological innovation, the more likely the applicant will choose the EPO as its ISA.

## Variables and estimation methodologies

We employ several categories of explanatory variables, representing each of the above hypotheses, in probit analyses specifying the probability of capture of a cited patent in a previous ISR as the binary dependent variable (*found_in_ISR*). The unit of analysis is the pair of citing and cited international families, both consolidated at the INPADOC family level.

For H1, we define three variables: *euro_cited* (cited family has its first priority, i.e., the earliest date, in EPC contracting states within a family, derived from tls201 and tls219 tables of PATSTAT), *us_cited* (cited family has its first priority in the US), and *jp_cited* (cited family has its first priority in Japan). When a cited family has its origin in the same region where an ISR is issued, we expect the ISA of the region to have a geographical advantage over the relevant technology. The expected sign is positive for each region, e.g., positive *jp_cited* coefficients for applications originating from Japan.

For H2, we define the citation lag between the first priority of a citing family and that of a cited family as *fam_cite_lag* (derived from tls201 and tls219 tables of PATSTAT). The longer the lag, the more easily the prior art is found at the time of the ISR. Therefore, its expected sign is positive.

For H3, we define *self* as a binary variable taking a value of one if one of the patents in a cited family and one of the patents in its citing family belong to the same applicant, based on PATSTAT (tls207) combined with EEE-PPAT, using “L2” identifier. Here we hypothesize the patent office will find it easier to locate prior relevant art within the same applicant. Therefore, the expected sign is positive.

For H4, we define *fwd_cite_of_the_cited* obtained from PATSTAT (tls212) as the number of forward examiner citations at the publication level (but consolidated at the family level), and made out to the cited patent family. When a prior art has been already cited by many patents, patent offices will find it easier to identify. Therefore, its expected sign is positive.

For H5, we first use a scope indicator, where *IPC4_count* is the total net count of IPC subclasses (4-digit IPC, derived from tls209) assigned in a citing INPADOC family. Because the patent classification of an application may change during the prosecution process, in both the international and national phases, we include all IPC subclasses to capture the breadth of a family. The number of claims of a patent correlates with the complexity of the technological content. As an indicator of the number of claims, we obtain *publn_claims_max_tls211*, which is the maximum number of claims registered on PATSTAT (tls211 table) in a citing INPADOC family. We do not simply rely on claims data from a single office, such as the EPO, because an application can be modified internationally during its prosecution. We also employ *invt_nr*, the maximum number of inventors in an application included in a citing INPADOC family, from PATSTAT (tls207). The size of the international family, *family_size*, is a count variable of applications in different countries in a citing INPADOC family (tls211/219). Because all of the complexity measures act negatively against prior art searches by patent offices, the expected signs are all negative.

In addition to the above variables used to test our hypotheses directly, we define two variables representing the capabilities of applicants in order to address the self-selection of ISA by the applicants. The first of these is *total_count*, which is the number of total applications that an applicant has made, taken from EEE-PPAT. The second is *applicant_avg_cited*, which is the number of average forward citations an applicant has received for an application, as calculated by PATSTAT (tls212) and EEE-PPAT. Both are supposed to represent the experience level of the applicant, and thus are used as instrument variables for instrumented PROBIT for the variable *ISA_changed.* This binary variable *ISA_changed* indicates that a US or Japanese applicant chose the EPO as their ISA (the EPO can be chosen by a US or Japanese applicant, but not vice versa for a European applicant). We obtain this information for PCT applications on PATSTAT, given the citation table tls212 has a field for citation origin whereas ISR is shown for PCT applications. Given that the first application country (RO) in a family is available from tls201, we can code the switch from RO to a different ISA. The correlation coefficient between *ISA_changed* and the dependent variable *found_in_ISR* is low, at around 0.03.

Lastly, we specify control variables for the originating area, being *JP_app* and *US_app* (applications from Japan and the US, respectively). We control for technology class using 35 WIPO technology classification dummies, setting the last classification as the reference class.

## Estimation results

The results under Model 1-1 in Table 1 employ the sample only from EPO regions. H1 is supported by the positive sign of *euro_cited* and the negative signs of *us_cited* and *jp_cited*. Likewise, the results for this model support H2, H3, H4, and H5, except that the estimated coefficient for the number of inventors has a positive sign, contrary to our expectation from H5. Model 1-2 further limits the sample to those from the EPO region and non-self-citations as a robustness check. The results are unchanged from Model 1-1. Model 1-3 employs all triadic samples from the EPO, USPTO, and JPO regions, with *JP_app* and *US_app* as applicant region controls, implying that the EPO is the reference category. The coefficients for the two region controls have negative and significant signs, indicating that ISRs prepared by the USPTO and the JPO are disadvantaged on average, compared with ISRs by the EPO. The binary variable *ISA_changed* indicates when US applicants or Japanese applicants select the EPO as their ISA. The estimated coefficient for *ISA_changed* is positive and significant, meaning that switching an ISA from the USPTO or the JPO to the EPO has made an ISR more complete. The results for the other variables are mostly unchanged from Model 1-1 and Model 1-2, except that the coefficient for the number of inventors has lost significance. The coefficient for *jp_cited* has shifted from a negative to a positive sign, but this is because of the pooled sample. This suggests that prior arts from the JPO area are easier to be found by the trilateral offices on average. The results from Models 1-1 and 1-2 clearly show that the EPO finds it more difficult to detect prior arts from the JPO area (See Tables 2, 3, 4 for summary statistics, definitions and correlation matrix).

**Table 1:** PROBIT analyses on the probability of ISR coverage; dep. var. = *found_in_ISR*

Model and sample	Model 1-1 (EP_app only)	Model 1-2 (EP app and non-self only)	Model 1-3 (EP, US and JP apps all pooled)
Method	Probit	Probit	Probit
ISA_ *changed*			0.3096426**** (0.0066579)
euro_cited	0.207075**** (0.016451)	0.2196004**** (0.017633)	0.1419984**** (0.0080393)
us_cited	−0.0456823** (0.016363)	−0.0523266*** (0.017525)	−0.0620007**** (0.0078305)
jp_cited	−0.457428**** (0.0169483)	−0.4633691**** (0.0181224)	0.0393056**** (0.0082601)
fam_cite_lag	0.003277**** (0.0003825)	0.0025981**** (0.0003906)	0.0030127**** (0.000212)
self	0.0635864**** (0.0103293)		0.2091817**** (0.0047187)
fwd_cite_of_the_cited	0.0000356**** (0.00000785)	0.0000321**** (0.00000783)	0.0000359**** (0.00000321)
IPC4_count	−0.0116202**** (0.0026942)	−0.0106372**** (0.0028234)	−0.0165033**** (0.0013614)
publn_claims_max_tls211	−0.0142597**** (0.0004492)	−0.014192**** (0.0004704)	−0.0080901**** (0.0001942)
invt_nr	0.0071474*** (0.0023555)	0.009076**** (0.0025359)	0.0000932 (0.0011831)
family_size	−0.0064288**** (0.0012222)	−0.0074571**** (0.0013424)	−0.006626**** (0.0007439)
JP_app			−0.0667862**** (0.0069462)
US_app			−0.2808785**** (0.0072769)
tech_field1	0.1446844*** (0.0447274)	0.1499931*** (0.0462607)	0.1444009**** (0.0271212)
tech_field2	0.1826226**** (0.0500017)	0.1890902**** (0.0511893)	0.0698306* (0.0274406)
tech_field3	0.1663785*** (0.0529874)	0.1589762*** (0.054606)	0.0329559 (0.0286252)
tech_field4	−0.0212283 (0.0485813)	−0.0195436 (0.0498217)	−0.0591332* (0.0277592)
tech_field5	0.0396309 (0.0627064)	0.0327979 (0.0648507)	0.0154652 (0.0335855)
tech_field6	0.0762131 (0.0476598)	0.0771954 (0.048991)	−0.0270184 (0.0274349)
tech_field7	0.1203062 (0.1757166)	0.1112099 (0.1794752)	0.202257**** (0.0420252)
tech_field8	0.2287141**** (0.0542056)	0.2308582**** (0.0562138)	0.0823158*** (0.0278554)
tech_field9	0.3632714**** (0.0504529)	0.3818553**** (0.0526016)	0.2023969**** (0.0283424)
tech_field10	0.0869068 (0.0446431)	0.0865379 (0.0461657)	0.073116** (0.0275033)
tech_field11	0.573764**** (0.0684509)	0.5695354**** (0.0717487)	0.452349**** (0.0364449)
tech_field12	0.0436221 (0.0597322)	0.0402991 (0.0614565)	0.0208029 (0.0350831)
tech_field13	0.2541932**** (0.0457902)	0.2437701**** (0.0473539)	0.1369031**** (0.0270368)
tech_field14	0.6062752**** (0.0436612)	0.6254198**** (0.0456541)	0.5058929**** (0.0275027)
tech_field15	0.7705994**** (0.0507952)	0.7553919**** (0.0539247)	0.5908729**** (0.0289889)
tech_field16	0.7391506**** (0.0444679)	0.7145057**** (0.0466877)	0.5942508**** (0.0274072)
tech_field17	0.4043448**** (0.0447423)	0.4241324**** (0.0466949)	0.2743913**** (0.0278738)
tech_field18	0.7592531**** (0.0791369)	0.7672647**** (0.0839064)	0.4946623**** (0.0391235)
tech_field19	0.5697184**** (0.045845)	0.569902**** (0.0480957)	0.339838**** (0.0286062)
tech_field20	0.3680992**** (0.0490161)	0.3547626**** (0.0506313)	0.1884243**** (0.0290885)
tech_field21	0.3057705**** (0.0547929)	0.2878575**** (0.0573025)	0.2135224**** (0.0302265)
tech_field22	0.0804922 (0.1395774)	0.1227397 (0.1436416)	−0.0830575 (0.0682282)
tech_field23	0.1434451*** (0.048968)	0.1493542*** (0.0507068)	0.1351532**** (0.0297479)
tech_field24	0.1409634* (0.0554432)	0.152254** (0.0573317)	0.1049673*** (0.0324254)
tech_field25	0.0837243 (0.0491715)	0.0882502 (0.0509684)	0.0083187 (0.029498)
tech_field26	0.0327776 (0.0480379)	0.0359581 (0.0496099)	−0.0087836 (0.0295359)
tech_field27	0.1589274**** (0.0450578)	0.1717163**** (0.0466351)	0.0817932*** (0.0287871)
tech_field28	0.3042161**** (0.0528322)	0.3184216**** (0.0556424)	0.2153852**** (0.0298522)
tech_field29	0.3330521**** (0.0527045)	0.3400129**** (0.054884)	0.1929519**** (0.0304772)
tech_field30	0.2264897**** (0.0598906)	0.241079**** (0.0615502)	0.1368849**** (0.0342744)
tech_field31	0.0094726 (0.0447409)	0.0004919 (0.0465451)	0.0446885 (0.0287091)
tech_field32	0.0457021 (0.0436223)	0.0444188 (0.0451479)	0.0033423 (0.0282497)
tech_field33	0.0576639 (0.0678101)	0.0663571 (0.0692349)	0.0616716 (0.0388799)
tech_field34	0.1700641** (0.0608972)	0.1883208*** (0.063318)	0.1239305**** (0.034291)
tech_field35	(reference)	(reference)	(reference)
constant	−0.1400582*** (0.0460341)	−0.1352025** (0.0479984)	−0.2124146**** (0.028279)
Log pseudo-likelihood	−158,417	−135,935	−661,846
*N*	249,307	214,766	1,031,127
# of clustered citing families	26,078	25,318	97,125

Model and sample	Model 2-1 (US app only)	Model 2-2 (US app and non-self only)	Model 3-1 (JP app only)	Model 3-2 (JP app and non-self only)
Method	Probit	IV Probit	Probit	IV Probit
ISA_changed	0.380766**** (0.0074961)	1.35421**** (0.3121828)	0.2758815**** (0.0169662)	0.010949 (0.1314657)
euro_cited	0.1776262**** (0.0120059)	0.148418**** (0.025389)	−0.031025 (0.0160179)	0.0203394 (0.0174625)
us_cited	0.050351**** (0.0114757)	0.0777813**** (0.015989)	−0.3377195**** (0.0155267)	−0.2974986**** (0.0169034)
jp_cited	−0.4295359**** (0.0121628)	−0.3751167**** (0.0427645)	0.8054234**** (0.0151802)	0.8367819**** (0.0175193)
fam_cite_lag	0.0046464**** (0.000329)	0.0026492**** (0.0005495)	0.0023379**** (0.0004175)	0.0005303 (0.0004425)
self	0.1123806**** (0.0076398)		−0.1759722**** (0.0082345)	
fwd_cite_of_the_cited	0.0000573**** (0.00000437)	0.0000551**** (0.00000526)	−0.00000566 (0.00000781)	−0.00000566 (0.00000799)
IPC4_count	−0.0215867**** (0.0022476)	0.0099131 (0.0110926)	−0.0176023**** (0.002381)	−0.0170435**** (0.0026306)
publn_claims_max_tls211	−0.0094453**** (0.0002733)	−0.0081833**** (0.0010323)	−0.0029271**** (0.0003468)	−0.0033284**** (0.0004149)
invt_nr	−0.0058672*** (0.0018111)	−0.0089979*** (0.0026536)	−0.0007108 (0.002112)	0.0008906 (0.0023144)
family_size	−0.0053694**** (0.0011327)	−0.0138593**** (0.0024961)	−0.0142835**** (0.0021553)	−0.0091501*** (0.0032126)
tech_field1	0.2428436**** (0.0486241)	−0.0146199 (0.104442)	0.0558508 (0.052299)	0.0819208 (0.0592823)
tech_field2	0.1705577*** (0.0493154)	−0.0698391 (0.1006609)	−0.0889339 (0.052323)	−0.0670638 (0.0592019)
tech_field3	0.0491601 (0.0491757)	−0.1330145 (0.0858773)	−0.0105879 (0.0550479)	0.0107328 (0.0615837)
tech_field4	−0.051275 (0.047647)	−0.2180253** (0.0796503)	0.0289418 (0.0556962)	0.0609505 (0.0621146)
tech_field5	0.0793476 (0.0562173)	−0.1366787 (0.1099556)	−0.016325 (0.0639327)	0.0388485 (0.071582)
tech_field6	−0.0329754 (0.0475002)	−0.1670512* (0.0761786)	0.0185922 (0.0542866)	0.0406843 (0.0609839)
tech_field7	0.1822923*** (0.0611697)	0.3286845** (0.1177346)	0.301815**** (0.0778456)	0.2877073*** (0.0844477)
tech_field8	0.1597099*** (0.0485588)	−0.1225467 (0.1126863)	−0.015146 (0.0533185)	0.0062975 (0.0599288)
tech_field9	0.2777179**** (0.0500796)	−0.0917514 (0.1392015)	0.0778071 (0.053753)	0.1100494 (0.0601185)
tech_field10	0.1435841*** (0.0483644)	−0.1696981 (0.1150714)	0.0614314 (0.0539854)	0.0919096 (0.0601537)
tech_field11	0.4360864**** (0.0566237)	0.2004486 (0.1047643)	0.4875324**** (0.0778804)	0.4982929**** (0.0862923)
tech_field12	0.092297 (0.0577402)	−0.244581* (0.1239686)	−0.0532708 (0.0671496)	−0.0637358 (0.0721385)
tech_field13	0.1019339* (0.046934)	−0.1875399 (0.1069902)	0.1091174* (0.0545724)	0.1280565* (0.0607982)
tech_field14	0.4952246**** (0.0483835)	0.1383903 (0.1460763)	0.5494072**** (0.0566347)	0.6247042**** (0.0645541)
tech_field15	0.5244111**** (0.0489719)	0.3890508**** (0.0903527)	0.6803185**** (0.0597871)	0.714162**** (0.0675115)
tech_field16	0.4855671**** (0.0475861)	0.2599136* (0.1045442)	0.7830664**** (0.0563774)	0.8116951**** (0.0635088)
tech_field17	0.3765414**** (0.0499197)	−0.1800641 (0.2036643)	0.124376* (0.0545345)	0.1169751 (0.0610249)
tech_field18	0.4386131**** (0.0637165)	0.1800015 (0.1398228)	0.3238745**** (0.0699398)	0.3073437**** (0.0791576)
tech_field19	0.3128908**** (0.049961)	−0.1536425 (0.1698294)	0.1735532*** (0.057277)	0.199093*** (0.0632013)
tech_field20	0.2963152**** (0.053857)	0.0091053 (0.1195746)	0.0366946 (0.0545363)	0.0480973 (0.0611276)
tech_field21	0.3192602**** (0.0516942)	−0.0179226 (0.1381289)	0.0727353 (0.057422)	0.0690492 (0.0641752)
tech_field22	−0.0679129 (0.0983615)	−0.5369288** (0.1984481)	−0.1661115 (0.1859442)	−0.0750412 (0.2190826)
tech_field23	0.2447045**** (0.0510565)	−0.045901 (0.1199372)	0.0652941 (0.0579753)	0.0724272 (0.0653014)
tech_field24	0.1936245*** (0.0572679)	0.0493307 (0.0954071)	0.073069 (0.0609101)	0.1169597 (0.0672215)
tech_field25	0.0765811 (0.0517362)	−0.1360526 (0.0942277)	−0.1090352 (0.0570776)	−0.0940606 (0.0639862)
tech_field26	0.1624198*** (0.0531509)	−0.0905513 (0.1068294)	−0.127283* (0.0556927)	−0.103975 (0.0627597)
tech_field27	0.1813037*** (0.055923)	0.0724606 (0.0936175)	0.0177349 (0.0555072)	0.0953296 (0.0656141)
tech_field28	0.2468054**** (0.0519663)	−0.1588447 (0.1502847)	0.114324* (0.0566256)	0.1558597* (0.0629745)
tech_field29	0.2274386**** (0.0550812)	−0.1564595 (0.1523844)	0.0931103 (0.056731)	0.0832719 (0.0631814)
tech_field30	0.2590443**** (0.0636966)	0.0802455 (0.1164088)	−0.0191918 (0.0608549)	−0.0317799 (0.0671536)
tech_field31	0.1797807*** (0.0540232)	−0.0533882 (0.1014106)	0.0473454 (0.0552925)	0.0693065 (0.061321)
tech_field32	0.057358 (0.0546367)	−0.0956397 (0.0949013)	0.0048358 (0.0555261)	0.0423921 (0.062192)
tech_field33	0.1509299* (0.0664652)	−0.0736027 (0.1271391)	−0.0436442 (0.0680619)	−0.045587 (0.0741097)
tech_field34	0.1494443** (0.0570063)	−0.1447077 (0.117844)	0.1162017 (0.0657658)	0.1356205 (0.0727767)
tech_field35	(reference)	(reference)	(reference)	(reference)
constant	−0.4960139**** (0.0489832)	−0.8014725**** (0.1324103)	−0.6243859**** (0.0553713)	−0.6797762**** (0.0615108)
Log pseudo-likelihood	−276,135	−467,661	−197,428	−186,380
*N*	455,830	363,328	325,990	264,805
# of clustered citing families	41,074	38,066	28,973	28,099

Robust standard errors are in the parentheses (clustering on citing family)

Model 2-2 and 3-2 use “total_count” and “applicant_avg_cited” as instruments for “ISA_ changed.”

**** <0.001; *** <0.005; ** <0.01; * <0.05

**Table 2:** Summary statistics

Variable	Obs	Mean	SD	Min	Max
found_in_ISR	1,057,671	0.387615	0.487206	0	1
ISA_changed	1,057,671	0.276951	0.447492	0	1
euro_cited	1,057,671	0.192777	0.39448	0	1
us_cited	1,057,671	0.434949	0.495751	0	1
jp_cited	1,057,671	0.357832	0.479362	0	1
fam_cite_lag^a^	1,042,360	9.420731	8.568765	−5	50
self	1,057,671	0.140923	0.347942	0	1
fwd_cite_of_the_cited	1,057,671	76.10043	419.7362	1	21,950
IPC4_count	1,057,671	3.35843	1.919123	1	25
publn_claims_max_tls211	1,057,671	19.1512	16.08566	0	296
invt_nr	1,057,671	3.099957	2.137287	1	39
family_size	1,057,671	7.833797	3.451029	4	41
total_count	1,057,671	9898.959	24,339.66	0	115,208
applicant_avg_cited	1,001,720	0.832080	1.836592	0	84.75
JP_app	1,057,671	0.313767	0.464023	0	1
US_app	1,057,671	0.44353	0.496801	0	1

^a^Observations of citation lag being more than 50 years are dropped from the analysis because of a reliability question. As a result, the usable sample size at the family level reduced to 97,125 for Model 1-3 (full sample)

**Table 3:** Variables

found_in_ISR	A binary variable, indicating a cited patent being caught in the previous ISR
ISA_changed	ISA changed to EPO (PATSTAT)
euro_cited	cited patent has its first priority in EPC contracting states (PATSTAT)
us_cited	cited patent has its first priority in the US (PATSTAT)
jp_cited	cited patent has its first priority in Japan (PATSTAT)
fam_cite_lag	citation lag between the first priority of a citing family and that of a cited family (PATSTAT)
self	examiner citation within the same applicant (PATSTAT&EEE-PPAT)
fwd_cite_of_the_cited	# of forward examiner citations (sum in a family) in PATSTAT
IPC4_count	the total net count of IPC subclasses (4-digit IPC) assigned in an INPADOC family (PATSTAT)
publn_claims_max_tls211	# of claims (maximum in an INPADOC family on PATSTAT tls 211 table)
invt_nr	# of inventors (PATSTAT)
family_size	# of applications in the same international family (PATSTAT)
total_count	# of total application that an applicant has made (EEE-PPAT)
applicant_avg_cited	# of average forward citations that an applicant has received per its patent (PATSTAT&EEE-PPAT)
JP_app	JPO as RO (PATSTAT)
US_app	USPTO as RO (PATSTAT)

**Table 4:** Correlation matrix

		1	2	3	4	5	6	7	8	9	10	11	12	13	14	15
1	found_in_ISR	1														
2	ISA_changed	0.031	1													
3	euro_cited	0.0763	−0.0631	1												
4	us_cited	−0.0721	0.1867	−0.4016	1											
5	jp_cited	0.016	−0.1431	−0.3544	−0.6462	1										
6	fam_cite_lag	0.0187	−0.0021	0.07	0.0259	−0.0746	1									
7	self	0.0778	0.006	0.0354	−0.0214	0.0139	−0.1865	1								
8	fwd_cite_of the cited	−0.0008	0.0246	−0.0414	0.1222	−0.0903	0.0216	−0.0019	1							
9	IPC4_count	−0.0131	−0.0296	0.0021	0.002	−0.0024	−0.0244	0.0106	0.0148	1						
10	publn_claims_max_tls211	−0.123	0.1023	−0.0611	0.0972	−0.0527	−0.0455	−0.0179	0.0324	0.1018	1					
11	invt_nr	0.0201	0.0202	0.015	0.0086	−0.0186	−0.0344	0.0559	0.0106	0.0981	0.0744	1				
12	family_size	0.022	0.0558	0.114	0.0557	−0.1495	0.0415	0.0471	−0.0015	0.0916	0.0832	0.1262	1			
13	total_count	−0.0147	−0.106	−0.0907	−0.1094	0.1781	−0.0983	0.0221	−0.0028	−0.0571	−0.0135	0.0077	−0.1658	1		
14	applicant_avg_cited	0.0268	0.1515	0.0227	0.1262	−0.1475	−0.0269	0.0203	0.0372	0.0651	0.059	0.0569	0.1793	−0.159	1	
15	JP_app	0.0141	−0.3031	−0.1598	−0.2679	0.4046	−0.0593	0.05	−0.0364	−0.0115	−0.0896	−0.0145	−0.2677	0.4326	−0.2631	1
16	US_app	−0.0644	0.5958	−0.1046	0.343	−0.2697	−0.0092	−0.0398	0.0575	0.0082	0.197	0.0099	0.0653	−0.2782	0.2602	−0.6049

Model 2-1 uses applications from the US only, and all of the results are consistent with the hypotheses, except that *euro_cited* has a positive coefficient (European prior art seems to be easier for searchers in the US). Model 2-2 also focuses on the US, and limits the citation data to non-self-citations as a robustness check, while employing two instrument variables for the variable *ISA_changed* through instrumented probit (IV Probit). The results are almost unchanged from those discussed earlier. The only exception is that the estimated coefficient for *IPC4_count* has lost significance.

Model 3-1 uses only the sample of applications from Japan to examine the local bias of prior art searches in Japan. As expected by H1, *jp_cited* has a positive and significant sign, whereas *us_cited* has a negative and significant sign. The other variables display similar results as Models 1 and 2 and are consistent with our hypotheses, except that *self* has a negative sign and there are insignificant coefficients for prior arts from Europe, the number of forward citations to the cited patents, and the number of inventors. For Japanese applications, the coefficient for *ISA_changed* lost significance in Model 3-2, suggesting that the advantage provided by the ISA change from the JPO to the EPO is from applicant self-selection. However, we do not observe this effect for the US-only applications in Model 2-2.

Some of the results relating to the 35 WIPO technology classes are also noteworthy. The estimated coefficients for class 14 and particularly classes 15 and 16 have consistently positive signs. The WIPO field classification for 14 is “Organic fine chemistry,” 15 is “Biotechnology,” and 16 is “Pharmaceuticals” (Table 5). Those technological classes are known as discrete technologies, the patents for these technology classes generally have a higher economic value when compared with more complex technologies. Because applicants conduct relatively complete searches before filing applications in discrete technology classes, the prior arts on the ISRs are thought to be relatively complete.

**Table 5:** WIPO technology fields

Field_number	Field_name
1	Electrical machinery, apparatus, energy
2	Audio-visual technology
3	Telecommunications
4	Digital communication
5	Basic communication processes
6	Computer technology
7	IT methods for management
8	Semiconductors
9	Optics
10	Measurement
11	Analysis of biological materials
12	Control
13	Medical technology
14	Organic fine chemistry
15	Biotechnology
16	Pharmaceuticals
17	Macromolecular chemistry, polymers
18	Food chemistry
19	Basic materials chemistry
20	Materials, metallurgy
21	Surface technology, coating
22	Micro-structural and nano-technology
23	Chemical engineering
24	Environmental technology
25	Handling
26	Machine tools
27	Engines, pumps, turbines
28	Textile and paper machines
29	Other special machines
30	Thermal processes and apparatus
31	Mechanical elements
32	Transport
33	Furniture, games
34	Other consumer goods
35	Civil engineering

*WIPO* World Intellectual Property Indicators (2011, p. 181)

## Discussion and further development

The overall results are consistent with our hypotheses, suggesting the binding of examiners (and searchers working for the PTOs) by various kinds of distances, including the technological complexity of applications. These are not very surprising results, but are supported by a novel methodology for the first time. Examiners (unlike inventors) are required by law to find prior art from all over the world, but are naturally bound by obstacles to searching. Most prior studies using examiner citations do not incorporate these informational obstacles in the way of examiners, and the present study has proposed and implemented a methodology to determine the existence of barriers. As stated in the literature review, prior studies on the difference of examination outcomes between patent offices (Jensen et al. 2005; Webster et al. 2007, 2014) have not explicitly considered these issues. Taking the cost of prior art search into a grant rate comparison offers a potential way of extending the research envelope. However, as explained in the methodology and data sections, we must first address several limitations. In particular, the US data require filtering on citation categories, and augmentation with rejected (abandoned) applications. Further, the results including instrument variables suggest self-selection is evident, but only for the Japanese sample. There is a need for further scrutiny using updated data including additional attributes of both applicants and applications.

These results have important policy implications, especially as PPHs rely on earlier outcomes from other patent offices. Given that knowledge is locally concentrated because of agglomeration economies, a local patent office may have an advantage over other distant patent offices in finding relevant prior knowledge locally. This is also likely because local examiners are educated and employed locally and have access to up-to-date information in the local language. In other words, the physical distance between the location of an invention and the location of its relevant prior art is not independent of the probability of the prior art found by examiners (and searchers employed or contracted by patent offices). If we attempt to evaluate merit by combining the work done by more than one patent office, an efficiency question depends on how distant patent offices duplicate their efforts. Put differently, in order to justify a system of physically dispersed patent offices on the planet, rather than a unitary single patent office that searches and examines patent applications worldwide, we need to know how complementary the offices are in terms of their searching capabilities. This paper provides a preliminary step toward responding to this key policy question.

## Footnotes

1 An invention needs to be novel to be patentable. Novelty can be denied by any evidence that an invention is already known to the public and “…there are no restrictions whatever as to the geographical location where or the language or manner in which the relevant information was made available to the public (EPO Guidelines for Examination, Part G, Chapter IV—State of the art).” In contrast, while prior art denies the novelty of an invention, “competing art” may not, as competing art offers a solution to the same problem in a different way (“What is prior art?,” EPO webpage, accessed November 30, 2015, http://www.epo.org/learning-events/materials/inventors-handbook/novelty/prior-art.html). This paper focuses on prior arts that question patentability as they can lead to the rejection of patent applications.

2 Suppose an invention is an improvement to a manufacturing device for semiconductors. The invention can then involve technological components from optics, materials science, as well as electrical machinery. Given countries have different levels of technological advantages by field (OECD 2015), and because examiners who have been educated locally are influenced by local technological (dis-)advantages, it is easier for an examiner to find a relevant prior art in a technological field where the country of the examiner has a relative advantage. A prior art may then be left undetected, if the examiner’s country is disadvantaged in the field. It is as if an examiner has an informational “horizon”, a metaphor being a radar station installed on the seashore unable to detect a ship over the horizon. There are complex impediments to searching for prior art, however, unlike the mere physical curvature of the Earth’s surface, which impedes the detection of objects over the horizon with radar.

3 In 2005, the US share of world PCT applications was 34.8 %, followed by the EPC countries with 30.6 %, and Japan with 18.0 %. Korea, which received the most PCT applications outside the regions covered by the trilateral offices, accounted for 3.3 %. China, which directly followed Korea in 2005, represented 1.8 %.

4 A patent family is “…a set of patents taken in various countries to protect the same invention” (Dernis and Khan 2004). The INPADOC family indicates a broader definition in that it includes “…all the documents directly or indirectly linked via a priority document belong to one patent family,” according to the EPO’s definition (https://www.epo.org/searching/essentials/patent-families/inpadoc.html, accessed November 30, 2015).

5 As described in the “variables” section, we measure the capabilities of applicants by the number of total applications and the average number of forward citations. Ideally, the capability of an applicant to search relevant prior arts is measurable and controllable. However, because of questions relating to endogeneity, we do not incorporate this in our analysis.

6 The EPO has recently announced that PATSTAT will cover application data resulting in rejections in the US (PATSTAT User Day Meeting Minutes, November 2, 2015).

7 In addition, a recent agreement between the USPTO and the JPO allows applicants from the US to choose the JPO as their ISA from 2015 onwards.

8 The KIPO’s small share is partly because our sample is limited to EPO, USPTO, and JPO triadic applications between 2002 and 2005. More recently, the KIPO accounted for the third-largest share of ISR publications. In 2013, the EPO remained the most selected ISA, with 37.7 % of all ISRs issued, followed by the JPO with 20.7 %, the KIPO with 14.8 %, and the USPTO with 8.1 % (WIPO 2011, pp. 68–69).

9 “Which PCT International Search Authority (ISA) should I use?” Webpage inovia.com accessed July 12, 2014. http://info.inovia.com/2013/09/which-pct-international-search-authority-isa-should-i-use/#sthash.91J9FjDM.dpuf.
